# Impact of simulated acid rain on chemical properties of Nyalau series soil and its leachate

**DOI:** 10.1038/s41598-024-52758-1

**Published:** 2024-02-12

**Authors:** Mohamad Hilmi Ibrahim, Susilawati Kasim, Osumanu Haruna Ahmed, Mohd. Rashid Mohd. Rakib, Nur Aainaa Hasbullah, Md. Tariqul Islam Shajib

**Affiliations:** 1 Agrotechnology Programme, Faculty of Resources Science and Technology, Universiti Malaysia Sarawak, 94300 Kota Samarahan, Sarawak Malaysia; 2 Department of Land Management, Faculty of Agriculture, Universiti Putra Malaysia, 43400 Serdang, Selangor Darul Ehsan Malaysia; 3 Universiti Islam Sultan Sharif Ali, Kampus Sinaut, Km 33 Jln Tutong Kampong Sinaut, Tutong, TB1741 Negara Brunei Darussalam; 4 Faculty of Sustainable Agriculture, Universiti Malaysia Sabah, 90000 Sandakan, Sabah Malaysia; 5 Division of Soil, Water and Environment, Care to People Denmark, 2400 Copenhagen, NV Denmark; 6 Present Address: Department of Natural Resources and Environmental Design, North Carolina Agricultural and Technical State University, Greensboro, NC USA

**Keywords:** Environmental sciences, Ecology

## Abstract

Greenhouse gases can cause acid rain, which in turn degrades soil chemical properties. This research was conducted to determine the effects of simulated acid rain (SAR) on the chemical properties of Nyalau series (*Typic paleudults*). A 45-day laboratory leaching and incubation study (control conditions) was conducted following standard procedures include preparing simulated acid rain with specific pH levels, followed by experimental design/plan and systematically analyzing both soil and leachate for chemical changes over the 45-day period. Six treatments five of which were SAR (pH 3.5, 4.0, 4.5, 5.0, and 5.5) and one control referred to as natural rainwater (pH 6.0) were evaluated. From the study, the SAR had significant effects on the chemical properties of the soil and its leachate. The pH of 3.5 of SAR treatments decreased soil pH, K^+^, and fertility index. In contrast, the contents of Mg^2+^, Na^+^, SO_4_^2−^, NO_3_^−^, and acidity were higher at the lower SAR pH. Furthermore, K^+^ and Mg^2+^ in the leachate significantly increased with increasing acidity of the SAR. The changes in Ca^2+^ and NH_4_^+^ between the soil and its leachate were positively correlated (r = 0.84 and 0.86), whereas the changes in NO_3_^−^ negatively correlated (r = − 0.82). The novelty of these results lies in the discovery of significant alterations in soil chemistry due to simulated acid rain (SAR), particularly impacting soil fertility and nutrient availability, with notable positive and negative correlations among specific ions where prolonged exposure to acid rain could negatively affect the moderately tolerant to acidic and nutrient-poor soils. Acid rain can negatively affect soil fertility and the general soils ecosystem functions. Long-term field studies are required to consolidate the findings of this present study in order to reveal the sustained impact of SAR on tropical forest ecosystems, particularly concerning soil health, plant tolerance, and potential shifts in biodiversity and ecological balance.

## Introduction

Acid deposition poses several threats to ecosystems by affecting plant health, diversity and structure, including processes and functions in the ecosystem^1,2^. Acid deposition is defined as accumulation of undesired chemical compounds in the atmosphere at toxic concentrations^3^. Acid deposits are materials (solids, liquids and gases) occurring in excess quantities from the average amount and present at the lowest layer of the atmosphere^4^. Acid deposition in the atmosphere can be attributed to diverse chemical compounds originating from fossil fuel combustion, agriculture, mining, and manufacturing activities. Acid deposition is a global threat that has been shown to result in various environmental and human health hazards such as depleting essential nutrients and increasing toxic metals, which can lead to reduced plant growth and biodiversity^5–7^.

Acid deposits refer to rain, snow, fog, particulates, and gases, whereas acid rain only refers to rainwater at pH below 5.6^8,9^. Acid rain mainly consists of sulfur dioxide (SO_2_) and nitrogen oxides (NOx) forming acidic compounds, whereas other greenhouse gases like Cl^−^ and CO_2_, linked to climate change and global warming. These gases undergo complex chemical reactions in the atmosphere after which they fall to the earth’s surface as wet or dry deposition^10^. According to Zhang et al.^11^, acid rain with a pH of 5.6 is deemed normal as atmospheric CO_2_ at a pressure of 101 kPa and temperature of 20 °C lowers rainwater pH from 7 to 5.6. This normalcy shifts when gases like N_2_O and SO_2_ contribute to a further decrease in pH below 5.6 due to increasing hydrogen ion concentrations.

Soil fertility and soil physico-chemical properties such as soil nutrients for plant growth and production, are commonly affected by prolonged exposure to acid deposition^12,13^. Several scientific reports have demonstrated that acid deposition may disrupt nutrient cycling in soil habitats, particularly by deteriorating soil physico-chemical properties, especially its fertility^14–17^. For example, soil nutrient leaching in White Mountain National Forest in the Central New Hampshire, US, resulted from acid deposition^18^. In addition, other studies on the impact of acid deposition on ecosystems have revealed that this phenomenon affects species richness and diversity^19,20^ and hydrological cycle, including water quality^21^.

More than that, this acidic precipitation lowers the soil pH, a process termed soil acidification. Research by Yang et al.^22^ shows that acidification leads to nutrient leaching, particularly of calcium and magnesium, while increasing the solubility and toxicity of metals like aluminum and lead. This results in reduced soil fertility and damage to plant root systems, adversely affecting plant growth and crop yields, as noted by Dai et al.^23^. Furthermore, soil acidification disrupts microbial communities, impacting critical processes like decomposition and nutrient cycling^24^.

Soil leaching is defined as the movement of nutrients from the upper soil profile to its lower depths^25^. Leaching typically causes soil pH to decrease with decreasing base cations concentrations. When acid deposition occurs, there is an increase in the solubility of heavy metals and Al mobilization in soils^26^. To this effect, accumulation of H^+^ ions reduces soil pH while increasing the solubility of heavy metals and Al mobilization. The leaching of macronutrients occurs due to the replacement of H + ions by acid rain, which increases soil acidity to levels that compromise fertility^27^. This phenomenon of soil acidification is not just theoretical; it has been observed on a large scale, for instance, in Southern China, where soil acidification was documented after 20 years of continuous exposure to acid rain^28,29^.

The mineral acid soils in Sarawak, Malaysia belong to four major series, namely Bekenu, Nyalau, Merit, and Stom series^30^. Nyalau series are the soils contaminated with eroded material from upslope areas with high content of sesquoxides^31^. According to Tan et al.^32^, Nyalau series belongs to *Typic paleudults*, therefore it is classified as acidic soils, with pH between 4.3 and 4.8 and CEC values below 24 cmol kg^−1^. The textural class of these soils is sandy clay loam with brownish yellow to yellow colouration. In Malaysia, the cumulative acid loading from the atmosphere to terrestrial ecosystems has been on the increase since 2010–2019^33^. As a result, SO_2_ and N_2_O composition in some states in Malaysia are 0.66 and 0.17 ppm, respectively^34^, while the pH of rainwater in selected industrial areas in Malaysia have reached 4.32^35^. EANET^36^ reported the annual rainwater pH at Petaling Jaya, Tanah Rata, Danum Valley, and Kuching, Malaysia as 4.15, 5.01, 5.21, and 5.43, respectively.

According to Department of Environment of Malaysia (unpublished data), the total SO_2_ emission in Malaysia was 0.25 ppm in 2020. Although this value is less than those of other countries, precautions should be taken to manage this occurrence to prevent it from increasing in severity. Although there are studies on simulated acid rain on soils in other areas^26,27,37,38^, there is dearth of information on the effect of SAR in Nyalau soils and its leachate. This study is important because the Nyalau series is not widely known. The Nyalau series, a tropical soil, is unique for its high sand content, strong acidity, and poor nutrient retention, making it challenging for agriculture but crucial for soil studies. Its characteristics and study are valuable for soil science and geology and contribute to our understanding of soil composition and geological history in certain regions facing the problem of acid rain.

This study embodies three objectives that significantly centre on the effects of simulated acid rain on chemistry and properties of Nyalau series (*Typic paleudults*) soil and its leachate. Firstly, the objective of the study is to identify the possibility of significant differences in soil fertility index and soil evaluation factor of Nyalau series soils when exposed to SAR. Secondly, the objective of the study seeks to ascertain the possibility of significant differences in the chemical properties of Nyalau series soils and its leachate when exposed to SAR. Finally, the study strives to examine the correlation and cluster between soil and leachate chemical properties across SAR pH. Soil fertility index and soil evaluation factor were used as key indicators to determine the effects of SAR on the fertility of Nyalau series.

## Materials and methods

### Soil collection, preparation and analysis

**Figure 1:**
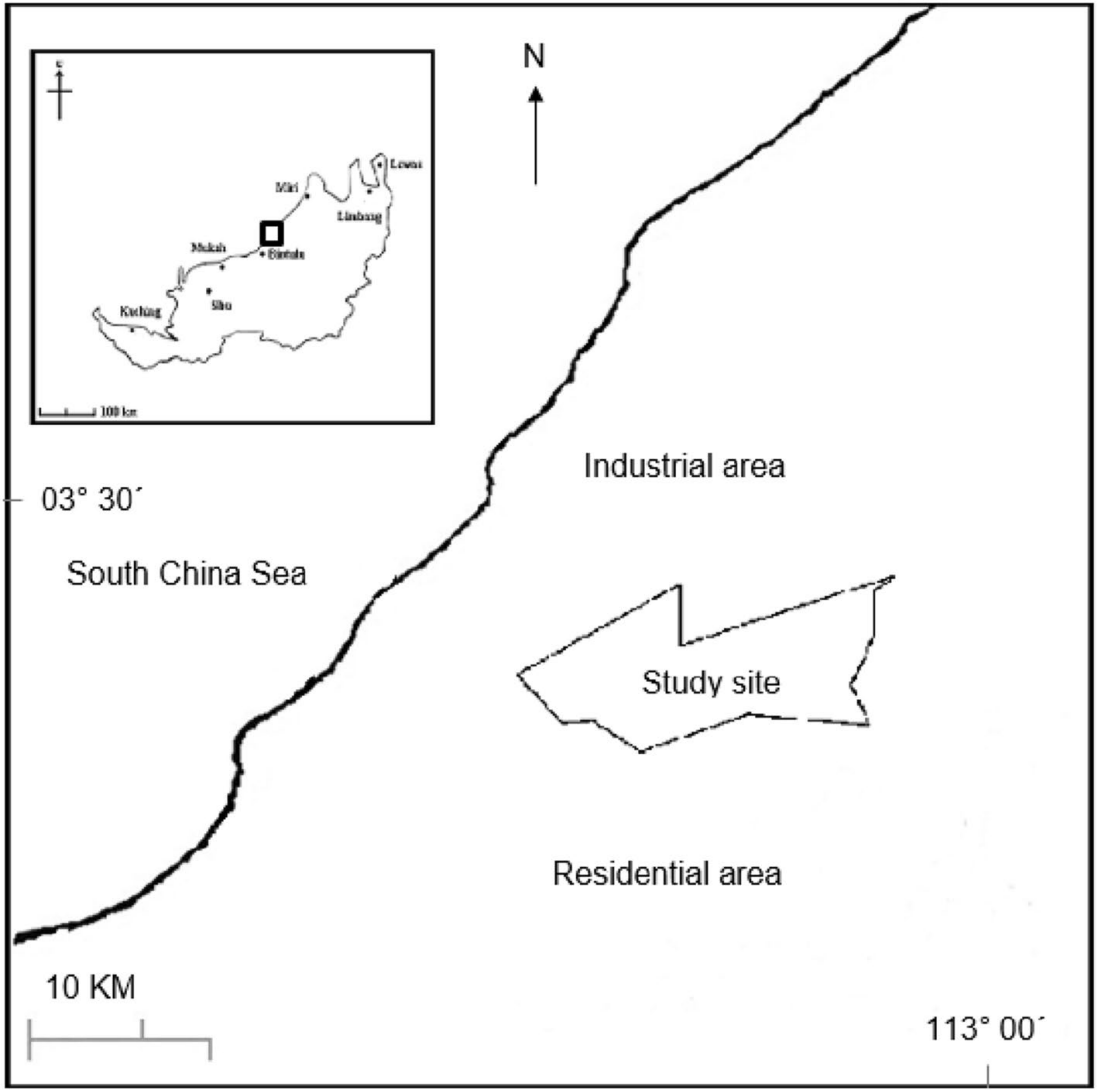
Location of the soil sampling sites in Universiti Putra Malaysia, Bintulu, Sarawak. Sampling were conducted ramdomly from several points in study sites.

The topsoil (0–20 cm depth) of Nyalau series from the undisturbed/minimal human intervention or alteration agricultural field, Universiti Putra Malaysia, Bintulu Campus, Sarawak, (03° 12.721′ N, 113° 4.477′ E) was collected from 10 points apart then bulked together using a spade until approximately 50 kg of soil (Fig. 1). The soil was collected in transparent plastic bags and transported to the laboratory, where it was air-dried in room temperature for a few days to a week and sieved to pass a 2 mm mesh. The initial chemical properties of the soil samples were determined using standard procedures as adopted from Tan^39^, for pH, Allen et al.^40^ for CEC, K^+^, Ca^2+^, Na^+^, Mg^2+^ and P, Keeney and Nelson method^41^ for NO_3_^−^ and NH_4_^+^, Rowell^42^, for acidity, Al^3+^, and H^+^ and Cheftetz et al.^43^ for soil organic matter and total organic carbon (Table 1).

**Table 1:** Selected soil physico-chemical properties of Nyalau series (*Typic paleudults*).

Variables	Value
pHwater	4.84
K^+^	0.54 mg kg^−1^
Ca^2+^	2.25 mg kg^−1^
Mg^2+^	0.02 mg kg^−1^
Na^+^	0.23 mg kg^−1^
Acidity	3.30 mg kg^−1^
Al^3+^	1.35 mg kg^−1^
H^+^	1.95 mg kg^−1^
CEC	10.2 mg kg^−1^
NH_4_^+^	49.04 mg kg^−1^
NO_3_^−^	21.02 mg kg^−1^
P	0.1 mg kg^−1^
SO_4_^2−^	18.0 mg kg^−1^
OM	6.0%

Data are expressed as the mean of three replications. Abbreviations: pHwater (pH in water); K^+^ (Exchangeable K); Ca^2+^ (Exchangeable Ca); Mg^2+^ (Exchangeable Mg); Na^+^ (Exchangeable Na); Acidity (Total acidity); Al^3+^ (Exchangeable Al); H^+^ (Exchangeable H^+^); CEC (Cation Exchange Capacity); NH_4_^+^ (Exchangeable NH_4_^+^); NO_3_^−^ (Exchangeable NO_3_^−^); P (Available P); SO_4_^2−^ (Available sulfate) and OM (Organic matter).

### Leaching experiment design and setup

The experiment was conducted using 18 polyethylene soil columns having 16 cm diameter and 28 cm depth and fitted with 26 holes (3 mm in diameter) at the bottom. The holes evenly distributed in a uniform circular pattern for optimal drainage. Analytical grade tissue paper was placed at the bottom of the column (to prevent soil loss) after which the column was filled with 270 g soil. Soil bulk density^44^ at the the undisturbed agricultural field site was first quantified, and the value was used to estimate the quantity of soil (i.e. soil without water content) to be used/ correspond with soil compaction in each column. This resulted in each empty soil column being filled with 270 g of air-dried soil, to simulate the natural condition of the Nyalau soil at the study sites. A tray was placed underneath each soil column to collect leachate.

### Treatment preparation and application

The soil in the columns were exposed to SAR by applying water with pH of 3.5, 4.0, 4.5, 5.0, 5.5, and 6.0.The pH 6.0 served as natural rainwater (control treatment). The selected SAR pH values of 3.5, 4.0, 5.0 and 5.5 were chosen to represent a range of acid deposition scenarios, from extreme to more moderate conditions enabling the study of soil responses under different environmental stress levels. A pH of 3.5 represents the worst-case scenario for acid rain worldwide and indicates the most severe environmental impacts. The other values, 4.0, 5.0 and 5.5, serve as projections ranging from extreme acidity to normal rainwater conditions. This range provides a comprehensive understanding of how different acidity levels can affect ecosystems, making the study relevant to real-world scenarios.

Water with varying pH levels was prepared by adding 0.1 molar H_2_SO_4_ and HNO_3_ in a 3:2 volume-to-volume ratio to distilled water, after which the pH was adjusted to the desired level^45^. The chemical properties of the SAR are presented in Table 2. Each treatment had three replications; thus, the total experimental units were 18. The experimental units were arranged in a completely randomized design (CRD) with aset up of 6 m × 4 m room having a 76% relative humidity and a temperature of 21 °C. Approximately 318 mL of SAR were applied to each soil column and this volume was based on the field capacity of the soil using a drip system operating at a flow rate of 2.71 mL s^−1^. The soil in the leaching columns were exposed to the SAR once every three days for 45 days (15 applications in total) at 8 pm. SAR application interval was based on average monthly/yearly rainfall events in Bintulu (MMD, Unpublished data), Sarawak, Malaysia. At the end of the experiment, the soil and its leachate were collected for chemical analysis.

**Table 2:** Chemical properties of simulated acid rain used in the incubation study. Data are expressed as mean of three replications.

Properties	Simulated acid rain (SAR) treatments					
	pH 6.0	pH 5.5	pH 5.0	pH 4.5	pH 4.0	pH 3.5
EC (µs cm^−1^)	15.62	7.14	11.95	26.67	46.87	130.53
Salinity (ppt)	0.02	0.02	0.02	0.02	0.03	0.08
TDS (mg L^−1^)	7.77	3.59	5.98	13.03	24.00	70.37
K^+^ (mg L^−1^)	0.17	0.3	0.36	0.53	0.97	2.2
Ca^2+^ (mg L^−1^)	1.74	0.62	0.72	1.01	0.7	1.51
Mg^2+^ (mg L^−1^)	0.07	0.04	0.06	0.07	0.05	0.09
Na^+^ (mg L^−1^)	0.33	0.2	0.39	1.06	0.27	0.75
NH_4_^+^ (mg L^−1^)	0.5	0.03	0.04	0.02	0.28	0.21
NO_3_^−^(mg L^−1^)	1.03	1.03	2.43	3.2	2.2	5.3
PO_4_^2−^(mg L^−1^)	0.32	0.31	0.25	0.2	0.22	0.22
S_2_^−^(mg L^−1^)	0.005	0.01	0.01	0.03	0.02	0.03
CI^−^ (mg L^−1^)	0.5	0.5	1.0	1.0	1.0	0.5
NO_2_^−^(mg L^−1^)	0.04	0.04	0.06	0.1	0.04	0.04

EC (Electric conductivity); Salinity (Salinity); TDS (Total Dissolve Solid); K^+^ (Exchangeable K); Ca^2+^ (Exchangeable Ca); Mg^2+^ (Exchangeable Mg); Na^+^ (Exchangeable Na); NH_4_^+^ (Exchangeable NH_4_^+^); NO_3_^−^ (Exchangeable NO_3_^−^); PO_4_^2−^ (Phosphate); SO_2_^−^ (sulfide); CI^−^ (chloride) and NO_2_^−^ (nitrite).

### Analysis of selected chemical properties of Nyalau series

After the incubation experiment, the soil samples in the columns were collected, air-dried, and sieved to pass through a 2 mm sieve for chemical analysis. The soil pH was measured in distilled water at a soil/water ratio of 1:2.5^39^. The CEC in mg/kg of the soil was determined using 1 M ammonium acetate buffered at pH 7. Exchangeable base cations were extracted using 100 mL of 1 M ammonium acetate buffered at pH 7, after which the filtrates were analyzed to determine the concentrations of exchangeable K, Ca, Na, and Mg using Flame Atomic Absorption Spectrometery (AAS) (iCE 300, Thermo Fisher Scientific®, NSW, Australia). The concentration of available P in the soil filtrate was determined using a UV–VIS spectrophotometer (UV-1800, Shimadzu, Kyoto, Japan) operated at 820 nm wavelength after extracting the soils using Bray’s solution (0.03 N of ammonium fluoride, NH_4_Fl in 0.025 N of HCI)^40^.

Soil available NO_3_^−^ and NH_4_^+^ were determined using Keeney and Nelson method^41^ followed by steam distillation^40^. Soil acidity, Al^3+^, and H^+^ were determined using the titration method^42^. The soil available sulfate was extracted using 0.5 M of NaHCO_3_, after which the extract was analyzed using ion chromatograph IC-MS (AI300, PerkinElmer Inc., USA). The loss-on-ignition (LOI) method was used to determine soil organic matter and total organic carbon^43^. A 5 g of oven-dried sample (dried at 6 °C for 24 h) was weighed into a porcelain dish, placed in a muffle furnace, and heated at 300 °C for 1 h to determine soil organic matter content.

The Soil Fertility Index (SFI; Eq. 1) and Soil Evaluation Factor (SEF; Eq. 2) of Nyalau series were calculated using the formulas of Moran et al.^46^ and Lu et al.^47^, respectively.

1 $$\begin{gathered} {\text{SFI }} = {\text{ pH }} + {\text{ organic matter }}\left( {\% ,{\text{ dry soil basis}}} \right) \, + {\text{ available P }}\left( {{\text{mg kg}}^{{ - {1}}} ,{\text{ dry soil}}} \right) \, \hfill \\ + {\text{ Exch}}.{\text{ K }}\left( {{\text{ceq kg}}^{{ - {1}}} ,{\text{ dry soil}}} \right) \, \hfill \\ + {\text{ Exch}}.{\text{ Ca }}\left( {{\text{ceq kg}}^{{ - {1}}} ,{\text{ dry soil}}} \right) \, + {\text{ Exch}}.{\text{ Mg }}\left( {{\text{ceq kg}}^{{ - {1}}} ,{\text{ dry soil}}} \right) \, - {\text{ Exch}}.{\text{ Al }}\left( {{\text{ceq kg}}^{{ - {1}}} ,{\text{ dry soil}}} \right) \hfill \\ \end{gathered}$$

2 $${\text{SEF }} = \, \left[ \begin{gathered} {\text{Exch}}.{\text{ K }}\left( {{\text{ceq kg}}^{{ - {1}}} ,{\text{ dry soil}}} \right) \, + {\text{ Exch}}.{\text{ Ca }}\left( {{\text{ceq kg}}^{{ - {1}}} ,{\text{ dry soil}}} \right) \, \hfill \\ + {\text{ Exch}}.{\text{ Mg }}\left( {{\text{ceq kg}}^{{ - {1}}} ,{\text{ dry soil}}} \right) \, \hfill \\ {-}{\text{ log }}\left( {{1 } + {\text{ Exch}}.{\text{ Al }}\left( {{\text{ceq kg}}^{{ - {1}}} ,{\text{ dry soil}}} \right)} \right) \hfill \\ \end{gathered} \right] \, \times {\text{ Organic matter }}\left( {\% ,{\text{ dry soil}}} \right) \, + { 5}$$

### Analysis of selected chemical properties of leachate

The leachate pH was measured using a pH meter (S220, Thermo Fisher Scientific®, USA) whereas electric conductivity (EC), salinity, and total dissolved solids were determined using EC meter (S70, Mettler Toledo Co., USA). Exchangeable cations were determined using AAS (AA5000, PerkinElmer Inc., USA) whereas nitrite (NO^2−^), phosphate (PO_4_^3−^), nitrate (NO_3_^−^), and ammonium (NH_4_^+^) were measured using UV-spectrophotometer (DR 2010, Hach©, USA).

### Statistical analysis

One-way analysis of variance (ANOVA) was used to detect between-treatment before after which treatment means were compare dusing Duncan’s New Multiple Range Test (post-hoc analysis) at *p* ≤ 0.05. Pearson’s correlation analysis was conducted to determine the relationship between the chemical properties of the soil and its leachate. In addition, Pearson’s correlation analysis was performed to analyze the response of soil and leachate variables across the pH of SARtreatment. The statistical analysis was performed using SAS version 9.4^48^.

Hierarchical cluster analysis (CA) was performed to find out similar groups of soil properties depending of origin (one soil type) and concentration. The CA was performed on the various chemical properties simulated acid rain and leachate, using a distance cluster between 15 and 20^49,50^. A distance criterion between two variables express how closely correlate within the group. Two cluster analyses by means of hierarchical dendrograms were performed by using SPSS 28.0 (IBM SPSS Statistics, USA) applied to the SAR and soil leachate. All these analysis collectively allowed for interpreting how SAR treatments affected soil and leachate composition, guiding conclusions on acid rain's impact.

## Results

### Effects of simulated acid rain treatments on soil properties

Soil pH, K^+^, SFI and SEF significantly decreased with increasing acidity of SAR. As example, significant decrease in soil pH and SFI (2.21% reduction) were recorded when the soil was exposed to SAR with pH 4.0 and pH 3.5. Potassium ions in the soil decreased from 0.037 to 0.019 mg kg^−1^ (48.64% reduction). Contrastingly, Mg^2+^, Na^+^, SO_4_^2−^, NO_3_^−^, and soil acidity significantly increased with increasing acidity of the SAR. Relative to control (natural rainwater) the soil which was exposed to SAR with a pH of 3.5 increased Mg^2+^, Na^+^, SO_4_^2−^, NO_3_^−^, and acidity by 193.33%, 101.30%, 46.2%, 18.65% and 22.02%, respectively. Furthermore, significant reduction was observed in the level of Al^3+^, H^+^, and Zn^2+^in soils exposed to SAR with pH 5.0. However, the K^+^, Ca^2+^ and Zn^2+^ cations decreased with increasing acidity of SAR (pH 4.0 and below). Similarly, available P in the soil significantly reduced from 1.62 mg kg^−1^ at SAR of pH 6.0 to 1.43 mg kg^−1^ at pH of 4.5, whereas SAR with pH 3.5 recorded an available P value of 1.57 mg kg^−1^. Furthermore, the Soil CEC, Ca^2+^, Fe^2+^, and NH_4_^+^ fluctuated across the SAR treatments whereas SEF generally remained unchanged (Table 3).

**Table 3:** Soil pH, chemical properties, soil fertility index (SFI), and soil evaluation factor (SEF) after exposed to simulated acid rain.

	Simulated acid rain (SAR) treatment					
	pH 6.0	pH 5.5	pH 5.0	pH 4.5	pH 4.0	pH 3.5
pH water	4.60 ± 0.03^a^	4.57 ± 0.04^a^	4.62 ± 0.07^a^	4.54 ± 0.01^a^	4.41 ± 0.03^b^	4.35 ± 0.03^b^
CEC (mg kg^−1^)	17.50 ± 0.71^a^	15.31 ± 0.51^b^	17.53 ± 0.62^a^	11.92 ± 0.88^c^	14.62 ± 0.48^b^	11.25 ± 0.56^c^
K^+^ (mg kg^−1^)	0.037 ± 0.001^a^	0.036 ± 0.003^ab^	0.032 ± 0.001^bc^	0.028 ± 0.001^c^	0.031 ± 0.001^c^	0.019 ± 0.001^d^
Ca^2+^ (mg kg^−1^)	0.97 ± 0.03^e^	1.55 ± 0.02^b^	1.44 ± 0.01^c^	1.34 ± 0.02^d^	2.08 ± 0.01^a^	1.57 ± 0.02^b^
Mg^2+^ (mg kg^−1^)	0.15 ± 0.01^d^	0.16 ± 0.008^d^	0.16 ± 0.003^d^	0.57 ± 0.01^a^	0.29 ± 0.01^c^	0.44 ± 0.01^b^
Na^+^ (mg kg^−1^)	0.77 ± 0.01^c^	1.53 ± 0.02^b^	1.51 ± 0.01^b^	1.66 ± 0.06^a^	1.51 ± 0.01^b^	1.55 ± 0.01^b^
Acidity (mg kg^−1^)	1.68 ± 0.35^c^	1.90 ± 0.08^ab^	1.72 ± 0.04^bc^	1.94 ± 0.06^a^	1.95 ± 0.05^a^	2.05 ± 0.06^a^
Al^3+^ (mg kg^−1^)	0.39 ± 0.01^a^	0.38 ± 0.02^a^	0.33 ± 0.01^b^	0.33 ± 0.01^b^	0.40 ± 0.01^a^	0.41 ± 0.01^a^
H^+^ (mg kg^−1^)	1.56 ± 0.03^a^	1.51 ± 0.07^a^	1.22 ± 0.03^b^	1.22 ± 0.01^b^	1.63 ± 0.02^a^	1.64 ± 0.05^a^
Fe^2+^ (mg kg^−1^)	0.012 ± 0.0006^b^	0.050 ± 0.001^a^	0.011 ± 0.0003^b^	0.011 ± 0.001^bc^	0.008 ± 0.003^c^	0.010 ± 0.001^bc^
Zn^2+^ (mg kg^−1^)	0.0041 ± 0.0001^a^	0.0033 ± 0.0001^b^	0.0024 ± 0.0001^c^	0.0027 ± 0.0001^c^	0.0034 ± 0.0001^b^	0.0032 ± 0.0002^b^
NH_4_^+^ (mg kg^−1^)	41.21 ± 0.46^c^	41.78 ± 0.52^c^	44.28 ± 0.50^b^	38.24 ± 0.50^d^	47.52 ± 0.51^a^	46.50 ± 0.44^a^
NO_3_^−^(mg kg^−1^)	32.12 ± 0.50f.	36.54 ± 0.06^e^	43.14 ± 0.25^c^	45.29 ± 0.46^b^	51.18 ± 0.33^a^	38.11 ± 0.38^d^
P (mg kg^−1^)	1.62 ± 0.04^a^	1.43 ± 0.05^a^	1.58 ± 0.03^a^	1.43 ± 0.03^b^	1.53 ± 0.01^ab^	1.57 ± 0.01^a^
SO_4_^2−^(mg kg^−1^)	7.10 ± 0.08^e^	7.16 ± 0.03^e^	7.82 ± 0.02^d^	9.45 ± 0.05^c^	12.60 ± 0.31^a^	10.38 ± 0.13^b^
SFI	12.21 ± 0.06^a^	12.15 ± 0.07^a^	12.20 ± 0.04^a^	11.98 ± 0.03^b^	11.95 ± 0.03^b^	11.94 ± 0.04^b^
SEF	19.16 ± 0.08^b^	19.24 ± 0.12b^b^	19.65 ± 0.10^a^	19.60 ± 0.07^a^	19.09 ± 0.04^b^	19.04 ± 0.08^b^

Values were expressed as means ± standard error, (n = 3). Different letters (^a, b, c and d^) within the same row indicate significantly different means at *p* ≤ 0.05 using a Duncan test. Abbreviations: pHwater (pH in water); CEC (Cation Exchange Capacity); K^+^ (Exchangeable K); Ca^2+^ (Exchangeable Ca); Mg^2+^ (Exchangeable Mg); Na^+^ (Exchangeable Na); Acidity (Total acidity); Al^3+^ (Exchangeable Al); H^+^ (Exchangeable H^+^); Fe^2+^ (Exchangeable Fe); Zn^2+^ (Exchangeable Zn); NH_4_^+^ (Exchangeable NH_4_^+^); NO_3_^−^ (Exchangeable NO_3_^−^); P (Available P); SO_4_^2−^ (Available sulfate); SFI (Soil Fertility Index); SEF (Soil Evaluation Factor).

### Effects of simulated acid rain (SAR) treatments on leachate properties

There was significant increase in K^+^ and Mg^2+^ concentrations in leachate as SAR levels were decreased from 4.0 to 3.5 (Table 4). K^+^ ions increased from 5.62 mg L^−1^ (SAR at pH 6.0) to 6.65 mg L^−1^ (SAR at pH 3.5) whereas Mg^2+^ ions increased from 0.72 mg L^−1^ (SAR at pH 6.0) to 0.83 mg L^−1^ (SAR at pH 3.5). The Na^+^ in the leachate significantly increased from 1.92 to 4.63 mg L^−1^ with increasing SAR acidity. The continued acidification reduced Na^+^ in the leachate to 2.68 mg L^−1^ (pH 3.5). The leachate of PO_4_^2−^ concentration did not significantly differences regardless of SAR pH. Other variables fluctuated across the SAR pH (Table 4).

**Table 4:** Leachate pH, and chemical properties after exposure to simulated acid rain.

	Simulated acid rain (SAR) treatments					
	pH 6.0	pH 5.5	pH 5.0	pH 4.5	pH 4.0	pH 3.5
pH	6.90 ± 0.06^ab^	6.70 ± 0.27^b^	7.08 ± 0.02^ab^	6.93 ± 0.04^ab^	7.28 ± 0.01^a^	7.07 ± 0.02^ab^
EC (µs cm^−1^)	38.57 ± 0.69^d^	47.27 ± 0.73^bc^	51.96 ± 0.47^a^	50.52 ± 0.37^a^	46.42 ± 0.32^c^	48.64 ± 0.61^b^
Salinity (ppt)	0.033 ± 0.007^ab^	0.040 ± 0.005^a^	0.013 ± 0.003^c^	0.023 ± 0.003^bc^	0.013 ± 0.003^c^	0.026 ± 0.003^b^
K^+^ (mg L^−1^)	5.62 ± 0.03^c^	6.07 ± 0.02^b^	5.71 ± 0.02^c^	5.71 ± 0.01^c^	6.13 ± 0.01^b^	6.65 ± 0.09^a^
Ca^2+^ (mg L^−1^)	12.94 ± 0.36^c^	13.66 ± 0.10^b^	13.70 ± 0.11^b^	12.58 ± 0.04^c^	14.22 ± 0.014^a^	13.58 ± 0.12^b^
Mg^2+^ (mg L^−1^)	0.72 ± 0.003^c^	0.75 ± 0.012^c^	0.56 ± 0.005^d^	0.72 ± 0.009^c^	0.96 ± 0.015^a^	0.83 ± 0.015^b^
Na^+^ (mg L^−1^)	1.92 ± 0.04^e^	3.71 ± 0.009^b^	4.63 ± 0.15^a^	3.71 ± 0.02^b^	3.26 ± 0.02^c^	2.68 ± 0.09^d^
Cu^2+^ (mg L^−1^)	2.67 ± 0.04^c^	2.99 ± 0.07^b^	3.08 ± 0.02^b^	3.54 ± 0.11^a^	3.14 ± 0.009^b^	2.96 ± 0.04^b^
Fe^2+^ (mg L^−1^)	16.01 ± 0.72^a^	11.42 ± 0.29^d^	16.05 ± 0.08^a^	13.29 ± 0.09^b^	10.43 ± 0.28^e^	12.06 ± 0.08^c^
NH_4_^+^ (mg L^−1^)	0.44 ± 0.07^c^	0.56 ± 0.06^bc^	0.66 ± 0.07^ab^	0.49 ± 0.04^c^	0.67 ± 0.009^ab^	0.74 ± 0.04^a^
NO_3_^−^ (mg L^−1^)	2.62 ± 0.04^a^	2.70 ± 0.04^a^	2.58 ± 0.08^a^	2.39 ± 0.08^b^	2.08 ± 0.04^c^	2.41 ± 0.03^b^
PO_4_^2−^ (mg L^−1^)	0.36 ± 0.04^a^	0.43 ± 0.007^a^	0.36 ± 0.07^a^	0.40 ± 0.06^a^	0.42 ± 0.01^a^	0.42 ± 0.02^a^

Values were expressed as means ± standard error (n = 3). Different letters within the same row indicate significantly different means at *p* ≤ 0.05 using a Duncan test.

### Relationship between soil and leachate properties

**Figure 2:**
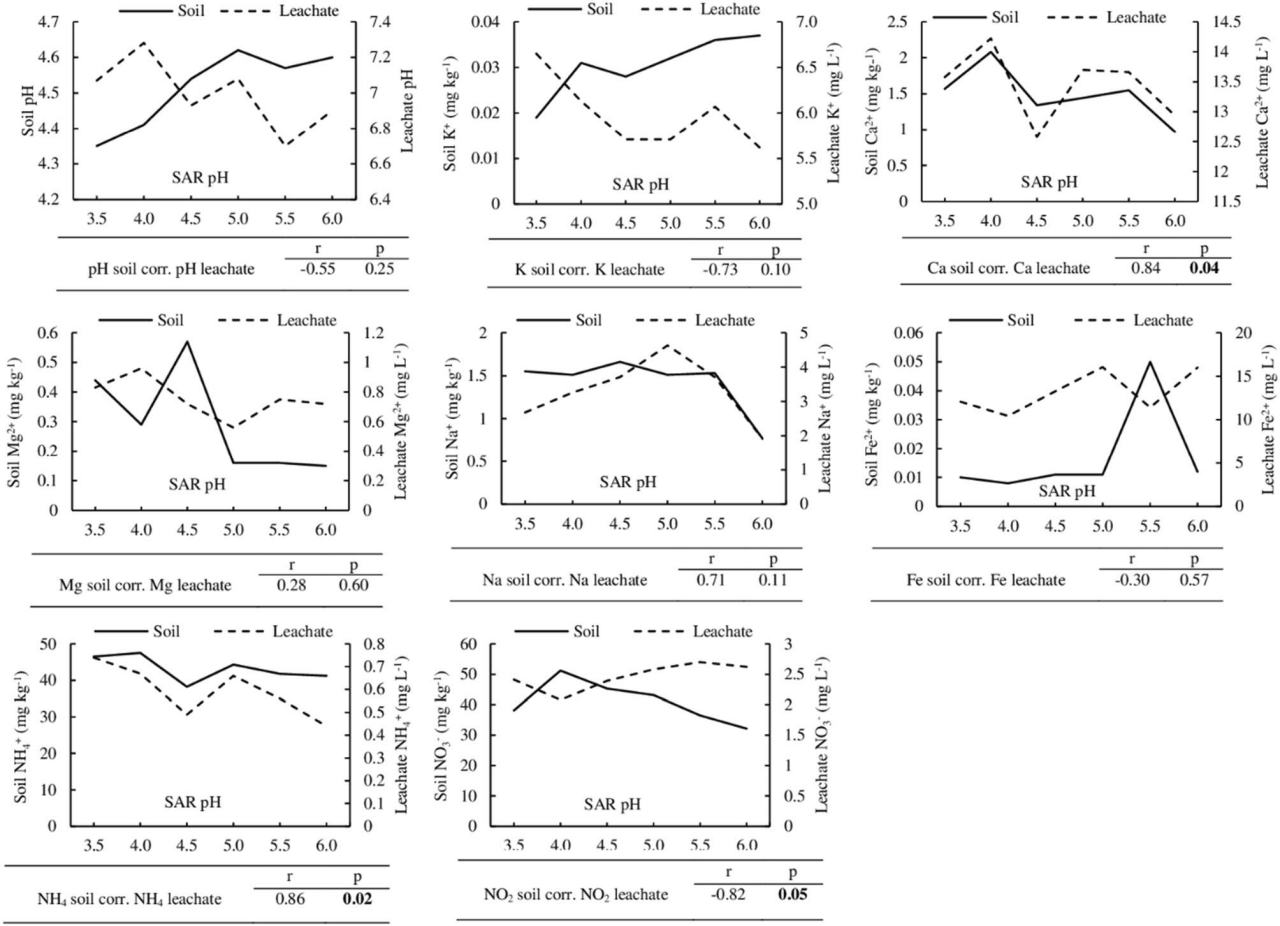
Trends of selected soil and leachate properties of Nyalau series (*Typic paleudults*) soil after exposure to simulated acid rain. Correlation analysis was conducted, and the relationship was indicated by the Pearson’s correlation coefficient (r) and probability level significant at *p* ≤ 0.05.

The relationship between the soil and its leachate properties was analyzed to determine acid deposition's effect on nutrients leaching or retention by the Nyalau series. The Pearson’s correlation analysis revealed that the changes in Ca^2+^ and NH_4_^+^ between the soil and its leachate positively correlated and the Pearson’s correlation coefficient (r) values were 0.84 and 0.86, respectively. However, the NO_3_^−^ in the soil and its leachate was correlated negatively (r = − 0.82). The correlation for the other variables were not significant (Fig. 2).

### Cluster analysis for soil and leachate properties

**Figure 3:**
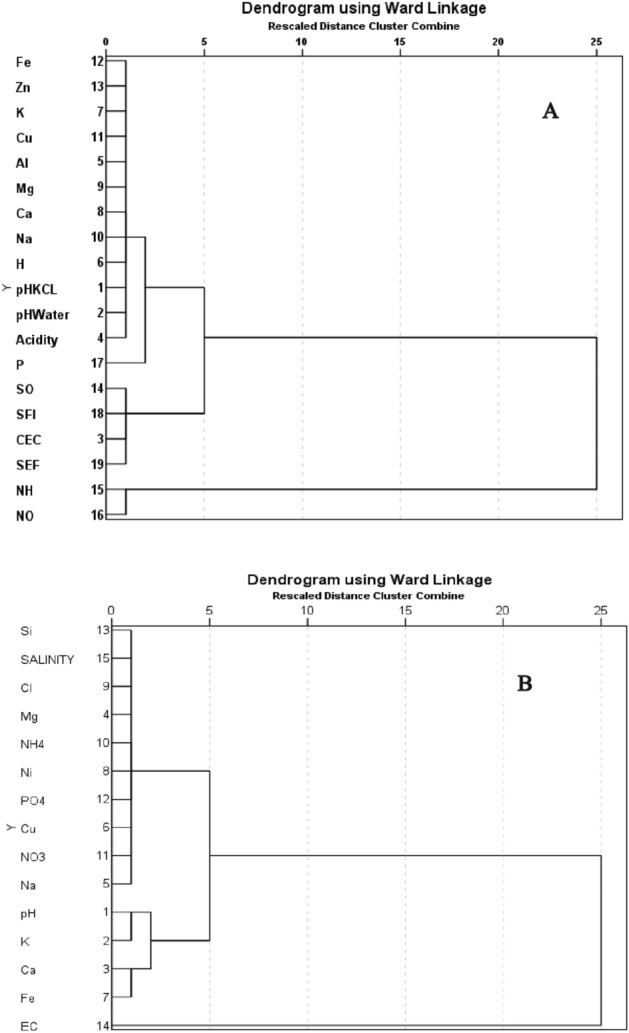
Hierarchical dendrogram for chemicals properties found in soil (**A**) and leachate (**B**) using Ward’s method.

The findings of CA are presented in two hierarchical dendrograms representing soil (Fig. 3A) and leachate (Fig. 3B). The dendrogram for soil comprise 3 clusters (Fig. 3A). NH_4_^+^ and NO_3_^−^ comprise first cluster and SO_4_^2−^, SFI, CEC and SEF comprise the second cluster and are associated with a low distance criterion around 1. The rest of the chemical properties acidity, pHwater, pH_KCl_, H^+^, Na^+^, Ca^2+^, Mg^2+^, Al^3+^, Cu^2+^, K^+^, Zn^2+^ and Fe^2+^ form the third cluster and they are associated in a very low distance at around 1. In Fig. 3B, the first cluster contains Cu^2+^,NO_3_^−^,PO_4_^2−^, NO_2_^−^, NH_4_^+^, Mg^2+^, Cl^−^, Salinity and S^2−^ and they are positioned at a very low distance around 1. In the second cluster, pH, K^+^, Ca^2+^ and Fe^2+^ form a group with a distance of CA below 3 whereas electrical conductivity (EC) is placed separately than cluster 1 and 2 with a high distance criteria at 25.

## Discussion

### Simulated acid rain and natural rainwater on soil properties of Nyalau series

Generally, the SAR treatments, including control, initially decreased soil pH (4.84). The pH ofsoilwith SAR pH below 4.5 (Table 3) was significantly low and this may cause reduction in the soil fertility index. Additionally, the soil exchangeable Al^3+^ and H^+^ were significantly increased because aluminium hydrolysis increases with increasing soil acidity. For example, a complete hydrolysis of one mole Al^3+^ ions produces three moles H^+^ ions to further decrease soil pH and this chemical reaction reduces soil CEC. This finding corroborates that of Zhang et al.^11^ who explained that acid rain increases soil acidity and H^+^ ions, leading to loss of mineral structure. Loss in mineral structure has been implicated in soil fertility decline. Wei et al.^51^ also reported that acid rain reduces soil fertility because it reduces soil pH and cation retention capacity.

Although soils have strong pH buffering capacity, the SO_4_^2−^, H^+^, NO_3_^−^, and NH_4_^+^ in acid rain favour the dominance of H^+^ ions on the soil exchange sites such that soil CEC is disproportionately dominated by hydrogen ions instead of base cations, especially K. Significant leaching of K^+^ in soil with SAR at pH 3.5 was expected due to the high acidity ofthis treatment. The dominance of stronger complementary adsorbed cations at the soil exchange sites could partly explain the loss of K into the leachate^26,51^. Acidic rainwater gradually diminishes exchangeable cations in topsoil because it facilitates changes in the nutrient pool and leaching of nutrients from the soil profile^44^. This observation is supported by Zhang et al.^11^, who reported significantly higher effluent K^+^ concentration of SAR at pH 3 and below.

The low SAR pH were responsible for low variations in Ca^2+^, Mg^2+^, Na^+^, acidity, NH_4_^+^, and SO_4_^2−^ in the soils compared with soil treated with natural rainwater. This finding is similar to that of Rampazzo and Blum^52^ who reported that exposing parent rock material to acid rain, inspite of having 30–80% calcite, reduced CEC and base saturation, particularly Ca contents. This suggests the fertility and the overall productivity of soils will decline if they are exposed to acid deposition for a long time. A notable reduction in soil pH enhances the solubility of aluminium, consequently elevating the concentration of Al^3+^ ions in the leachate. This finding aligns with Mulder et al.^53^ observation, where they reported the phytotoxic effects due to increased dissolution of Al^3+^ in soil leaching experiments conducted in both the Netherlands and New Hampshire, USA.

Soil Zn^2+^ solubility has increase with decreasing pH (3.5–6.0) because the solubility of Zn decreases with increasing soil pH. High levels of soil contamination, with soluble Zn^2+^ reaching 19,570 mg/kg and Cu^2+^ up to 322.4 mg/kg^38^, enhance the phytoavailability of heavy metals^14^, leading to increased uptake by plants. The very acidic SAR treatments increased soil exchangeable sulfate 46.20% because of sulfate adsorption to form sulfuric acid which upon decomposing, releases H^+^ and SO_4_^2−^ ion. This reaction occurs at low soil pH^54^. Soil available ammonium increase with increasing acidity of SAR. The increase in NH_4_^+^ concentration is consistent with the report of Johnson et al.^55^, who demonstrated that acid rain increases nitrogen mineralization and nitrification in forest soils.

### Simulated acid rain and natural rainwater on leachate properties of Nyalau series

Leachate pH was highest with lower SAR pH compared with natural rainwater. According to De Walle et al.^56^, the increase in leachate pH was due to the accumulation of base cations, especially Ca^2+^ and Mg^2+^ (Table 4). This result also explains the movement of Ca^2+^ and Mg^2+^ down the soil profile, corroborating the results of Zhang et al.^11^ on Latosol of Southern China. Low electrical conductivity and salinity values were recorded with lower SAR pH because the accumulation of base cations in the leachate increased the EC of the soil. The base cations in the leachate of the lower SAR pHs were higher than with the natural rainwater (Table 4). This present study suggests that acid rain causes leaching of the bases and this could cause ground water pollution through enrichment through lost nutrients from the soil profile.

### Overall implication of varying simulated acid rain on soil and leachate properties

The incubation of Nyalau soil series with SAR generally had negative effects on pH, K, Fe and NO_3_ of the soil and its leachate. This includes a decrease in soil pH, indicating increased acidity, and reductions in the concentrations of potassium (K), iron (Fe), and nitrate (NO_3_) in the soil. The results indicate that when the pH of SAR decreases from 6.0 to 3.5, the pH and potassium (K) content in the soil and leachate also decrease. This is confirmed by the data in Tables 3 and 4. The increased soil acidity with the low pH SAR is related to high H^+^ concentration. The accumulation of H^+^ from acid deposition increased the soil acidity^27,51^. Increase in the soil acidity through acid deposition might have affected the solubility of heavy metals such as Fe, as observed in the soil with low SAR pH. Furthermore, acidic pollutants can cause P fixation by Al and Fe in soils^57^ and this explain low available P content in this present study (Table 3). The positive relationship between soil and leachate for Ca^2+^ and NH_4_^+^ was due to insufficient time (45 days) for leaching of cations from the soil. This slower leaching rate is due to the complex interplay of physical, chemical, and environmental factors within the soil. Essentially, these ions are not as readily mobilized or washed out of the soil compared to other elements, indicating a delayed response to the leaching process influenced by soil composition and conditions^58^.

More than that the similarities of SO_4_^2−^, SFI, CEC and SEF in hierarchical dendrograms of soil have shown that the fertility of Nyalau series soil have also influenced by SO_4_^2−^. We believe it was happening because of the presence of sulphuric acid (H_2_SO_4_) from SAR treatments. Our argument is consistent with finding in Table 3 recorded higher SO_4_^2−^ content under low SAR pH treatments. Similar study reported by Hüttl and Frielinghaus^59^ in Eastern, Germany who shows that air pollutant or acid rain content with H_2_SO_4_ could reduce the soil fertility accelerating soil acidification. In the leachate hierarchical dendrograms, there are similarities of soil water pH, K and Ca. This results reliable comes from accumulation of base cation while exposure to SAR as discussing in previous section.

### Management implication of simulated acid rain on soil and leachate properties

Even with a short incubation study (45 days), we found a 2.21% reduction in the fertility of Nyalau series and 5.43% reduction in soil acidity as compared when exposed to natural rainwater (control treatment). The lower SFI of the soil in the present study (11.94) compared with research on a secondary forest in Lundu, Sarawak, by Perumal et al.^60^ where SFI of 19.63 was recorded, indicates the prolonged negative impact of acid rain on soil fertility. These results showed that acid rain impacted soil and leachate properties, and it is possible that prolonged acid rain exposure will further modify soils of the Nyalau series detrimentally.

Therefore, for a comprehensive understanding of acid rain's effects, a long term study, possibly over a year, is recommended. This allows for observing long-term ecological and soil changes. Complementing this with advanced modeling would provide a holistic view, predicting future impacts and aiding in effective environmental management strategies, crucial for sustaining ecosystems and agricultural productivity in the face of environmental changes. Therefore, understanding the prolonged impacts of acid rain on soil properties is not only an ecological necessity but also crucial for human sustainability.

## Conclusion

The study focused on the impact of simulated acid rain (SAR) on the Nyalau series soil, examining a range of acidity levels from less acidic (pH 5.5 and 5.0) to more acidic (pH 4.0 and 3.5). It was found that with increasing acidity, especially at pH 3.5, the soil experienced significant changes: a decrease in pH, potassium, and fertility, and an increase in magnesium, sodium, sulfate, nitrate, and overall acidity. The leachate from the soil also showed increased levels of potassium and magnesium, indicating a leaching effect that could lead to nutrient deficiencies for plants. The study also noted a positive correlation between changes in calcium and ammonium levels in both soil and leachate, and a negative correlation in nitrate levels, highlighting complex interactions between soil acidity and nutrient dynamics.

The results of our study have important practical implications for both land management and environmental policy. Land managers are suggested to regularly conduct comprehensive soil health assessments, especially in areas vulnerable to acid rain or soil acidification. These assessments should go beyond simply measuring pH and consider chemical properties such as K^+^, Mg^2+^ and NO_3_^−^ to inform soil treatment plans. In terms of policy, the observed deleterious effects of acidic treatments on soil properties call for stricter pollution regulations to curb acid rain, and the data could further guide the establishment of safe areas for agriculture and forestry based on the resilience of soils to acidification.

## Data availability 

The datasets generated and/or analysed during the current study are available from the corresponding author upon reasonable request.
